# “Study protocol for the ≥65 years NOrthern jutland Cohort of Fall risk Assessment with Objective measurements (the NOCfao study)”

**DOI:** 10.1186/s12877-020-01535-6

**Published:** 2020-06-08

**Authors:** Morten Villumsen, Bo Grarup, Steffan Wittrup Mc Phee Christensen, Thorvaldur Skuli Palsson, Rogerio Pessoto Hirata

**Affiliations:** 1 Department of Elderly and Health, Section of Training and Activity, Aalborg Municipality, Aalborg, Denmark; 2 Department of Health Science and Technology, Aalborg University (AAU), Aalborg, Denmark; 3 Department of Physiotherapy, University College of Northern Denmark (UCN), Selma Lagerløfs Vej 2, 9220 Aalborg East, Denmark; 4 Performance and Technology, Department of Health Science and Technology Aalborg University, Niels Jernes Vej 12, 9220 Aalborg East, Denmark

**Keywords:** Technical measurements, Accelerometry, Physical activity, Elderly, Physical exposures, Risk prediction, Fall detection, Older people, Physical behavior

## Abstract

**Background:**

Accidental falls are common among community-dwellers, probably due to the level of physical activity and impaired postural stability. Today, fall risk prediction tools’ discriminative validity are only moderate. In order to increase the accuracy, multiple variables such as highly validated objective field measurements of physical activity and impaired postural stability should be adressed in order to predict falls. The main aim of this paper is to describe the ≥65 years NOrthern jutland Cohort of Fall risk Assessment with Objective measurements (NOCfao) investigating the association between physical activity and impaired postural stability and the risk of fall episodes among community-dwelling older adults.

**Methods:**

The study consists of a baseline session where the participants are asked to respond to three questionnaires, perform physical tests (i.e., measuring strength in the upper and lower extremities, balance, and walking speed), participate in an assessment of pain sensitivity, and to wear an ankle mounted pedometer for measuring physical activity for 5 days. Subsequently, the fall incidences and the circumstances surrounding the falls during the previous 1 to 2 months will be recorded throughout a one-year follow-up period.

**Discussion:**

This study will add to the present-day understanding of the association between physical activity and impaired postural stability and the risk of fall episodes among community-dwelling older adults. These data will provide valid and reliable information on the relationship between these variables and their significance for community-dwelling older adults.

**Trial registration:**

ClinicalTrials.gov identifier: NCT2995317. Registered December 13th, 2016.

## Background

Accidental falls are common in the elderly population [1] with increased risk of falls with increased age and level of fragility [2–5]. Among home-dwelling older adults (≥65 years), 33% will have at least one fall per year [2, 6, 7] with higher prevalence as the age increases (i.e., 40% over 80 yrs) [8–10]. Such accidents cause injuries, fear, and reduced quality of life [11, 12] along with increased morbidity (e.g., higher risk of institutionalization and hospitalization) and mortality [11–13]. Moreover, falls are the most prevalent cause for injury-related deaths among older adults ≥65 years [14]. It is estimated that up to 0.20% of the gross domestic production and 0.85–1.50% of the health care costs are related to fall incidences [15]. With a rapidly growing proportion of individuals over 65 years, this group is projected to represent 15.6% of the world’s population by 2050 [16] constituting both a present and future societal challenge. In summary, the burden of accidental falls among older adults on the individual, family, community, and society is tremendous [2]. Therefore, fall prevention in this population is considered a point of focus in relation to public health globally [17].

Various risk factors, such as muscle weakness, history of falls polypharmacy, gait, and balance deficits increase fall risk [18–20], indicating that both clinical data and technical assessments are important for screening a multifactorial problem such as fall risk [6, 21]. An embedded definition by Tinetti and colleagues (1998) states a fall as: “*an event which results in a person coming to rest unintentionally on the ground or lower level, not as a result of a major intrinsic event, such as stroke, or overwhelming hazard*” [11]. Accidental falls are often classified into intrinsic (e.g. non-accidental causes such as history of falls, age, gender, solitary lifestyle, fear of falling, nutritional deficiencies, cognitive disorders, attenuated vision, balance and gait impairment, and foot problems), extrinsic (e.g. polypharmacy) and environmental (e.g. lighting, footwear, and bumpy and slippery surface areas) risk factors [18, 19, 22, 23]. Intrinsic risk factors such as poor balance or gait impairment are particularly common causes to ‘slip and trip’ which may result in accidental falls among older adults [9, 22, 24, 25].

In general, fall prevention in the older adult population may be divided into three key points; 1) screening methods for identifying individuals at high risk of falling, 2) determining the multiple risk factors for a fall, and lastly, 3) implementing individualized interventions into clinical practice [1, 26]. Numerous studies have proposed different screening tools for fall risk assessment [23, 26–28]. The majority of guidelines on fall risk screening usually include a combination of questionnaire-based screening tools (e.g., fall history, walking difficulties, and balance deficits) and functional tests targeting balance and gait impairments [29]. In order to increase the accuracy, a successful screening tool should consist of multiple variables in order to predict falls [1, 30].

Despite the development of numerous fall risk prediction tools among community-dwelling older adults, the discriminative validity to identify those at risk of falling are only moderate [31–34], and a recent study even found disagreement for screening the risk of falling in older adults between several commonly used fall risk assessment methods [35].

There are indications that physical function and activity are closely associated with falls among older adults [36]. Moreover, there are some indications of the existence of a u-shaped pattern in the level of physical activity and risk of falls where both low and high levels of physical activity are associated with greater risk of falls [22]. This highlights physical activity as a key explanatory variable to falls [36]. Previous studies have used accelerometers to investigate the association between physical activity and falls [37, 38]. However, the technologies used (Activpal and Actigraph) have shown low criterion validity compared to hand tally [39] and Stepwatch [38], respectively. This may be due to the low walking speeds represented in these populations [40], which may constitute issues in data interpretation and thereby questioning the research outcome. In this regard, addressing the exposure of time using highly validated, objective diurnal field measurements of physical activity may provide or alter the conventional associations [36, 41] which have not been addressed in the above-mentioned studies [31–34].

Although multiple factors increase the risk for an accidental fall, deficits in postural stability during gait and balance tasks present especially high odds ratios {OR [Range]) (gait: (2.9 [1.3–5.6]), balance (2.9 [1.6–5.4])}, only exceeded by muscle weakness (4.0 [1.5–10.3])) and history of falls (3.0 [1.7–7.0]) [19]. For example, a previous study demonstrated the predictive value of a decrease in walking speed, probably due postural instability, for indoor falls among older adults (Internal rate of return (IRR) = 1.86) [42]. Additionally, the control of lateral stability during standing was associated with increased risk of falls in an older adult population [43] although, to the knowledge of the authors, no reference cut-off values using postural sway measurements when evaluating fall risks have been proposed in the literature so far.

Despite the large amount of evidence relating falls to different parameters, most of the current validated and objective methods used for assessing fall risks are not easily implemented in clinical practice. A risk assessment tool should be both practical, simple, and feasible in terms of usability but also be highly sensitive to distinguish between those at high and low risk of falling to ensure good discriminate validity and power [44]. The risk assessment tool should be valid and reliable for investigating risk factors for falls but also be based on objective measures of physical activity in combination with selected physical- and psychological risk factors, using a prospective design with reports of falls.

## Methods/design

### Aims

The main aim of this paper is to describe the ≥65 years NOrthern jutland Cohort of Fall risk Assessment with Objective measurements (NOCfao) by investigating the association between objectively measured physical activity and monthly prospective measures of falls over a one-year period among community-dwelling older adults ≥65 years. The ancillary clinical perspective of NOCfao is to develop a clinically applicable fall risk prediction tool (FRPT), based on the level of objectively measured physical activity and selected physical and psychological risk factors.

The main study questions of this prospective, observational cohort study are:

1. Is physical activity among community-dwelling older adults associated with risk of fall episodes?
2. Does impaired postural stability among community-dwelling older adults increase the risk of falls episodes?

### Study design and setting

This prospective, observational cohort study and data collection was registered in accordance to the current guidelines (FOU-UU-006) at the Danish Data Protection Agency, the local Ethics Committee (N-20160020), and registered at ClinicalTrials.gov (identifier:) NCT02995317. This study protocol on the NOCfao study complies to the SPIRIT statements for defining standard protocol items [45], including the recommendations for trials protocol submissions [46] and the STROBE guidelines for reporting observational studies [47]. The study, is led by the University College of Northern Denmark (Department of Physiotherapy), in collaboration with Aalborg University (Institute of Health Science and Technology) and Aalborg Municipality (Department of Elderly and Health) will be conducted in accordance with the principles of the Declaration of Helsinki [48]. The administrative information on the NOCfao study are given in Table 1.

**Table 1:** Administrative information on the NOCfao study

Section/item	ItemNo	Description
Primary Registry and Trial Identifying Number	1	ClinicalTrials.gov identifier: NCT02995317
Date of Registration in Primary Registry	2	December 13th, 2016
Secondary Identifying Numbers	3	The North Denmark Region Committee on Health Research Ethics (N-20160020) The Danish Data Protection Agency (FOU-UU-006)
Source(s) of Monetary or Material Support	4	The study is externally supported by the Trygfonden Foundation (ID: 119365)
Primary Sponsor	5	University College of Northern Denmark, Denmark
Secondary Sponsor(s)	6	Aalborg University, Denmark Aalborg Municipality, Denmark
Contact for Public Queries	7	Master of Rehabilitation, Bo Grarup; address: Selma Lagerløfs Vej 2, 9220, Aalborg East, Denmark; tel.: 0045 72,690,953; e-mail: bog@ucn.dk
Contact for Scientific Queries	8	Master of Rehabilitation, Bo Grarup; address: Selma Lagerløfs Vej 2, 9220, Aalborg East, Denmark; tel.: 0045 72,690,953; e-mail: bog@ucn.dk
Public Title	9	Who is Falling? – Fall Risk Prediction Among Community-Dwelling Elderly (NOCfao)
Scientific Title	10	The ≥65 Years NOrthern jutland Cohort of Fall risk Assessment with Objective measurements (the NOCfao study)
Countries of Recruitment	11	Denmark
Health Condition(s) or Problem(s) Studied	12	Incidence of falls
Intervention(s)	13	Not applicable
Key Inclusion and Exclusion Criteria	14	Inclusion criteria: home-dwelling, manage body transfer independently, walking ability for at least 10 m with or without assistive devices. Exclusion criteria: progressive neurological or rheumatologically illness, diagnosed vestibular problems, experienced pain in a substantial degree limiting or obstructing everyday living, known uncorrected visual or hearing problems, serious difficulties in speaking, understanding or reading Danish, or cognitive impairment equivalent to Mini Mental State Examination < 23
Study Type	15	Prospective, observational cohort study
Date of First Enrollment	16	December 2016
Target Sample Size	17	600
Recruitment Status	18	Recruiting: participants are currently being recruited and enrolled
Primary Outcome(s)	19	Self-reported number of falls confirmed by monthly phone calls [Time Frame: One year follow-up]
Key Secondary Outcomes	20	History of fall incidences within the past year; differences between objective measures and self-reported level of PA

The minimum amount of trial information (20 items) recommended by the World Health Organization (Version 1.2.1) [49].

### Study population and recruitment process

This study is conducted in Aalborg Municipality at different activity centers in collaboration with care workers, nurses, and staff at the activity centers, all employed at the Department of Elderly and Health, Aalborg Municipality. Various public advertising platforms are selected to draw attention to the study. Information material describing the study is posted in local newspapers, newsletters, social media, and through posters distributed to activity centers in Aalborg Municipality. Furthermore, the project manager arranges briefing meetings with groups of employees to provide clarification of the project regarding inclusion/exclusion criteria and information about the study procedure. Information material and informed consent templates will be distributed to those interested in supporting the recruitment process of the study population.

Participants are eligible for inclusion if they are aged ≥65 years, home-dwelling, able to manage body transfer independently, and with a walking ability for at least 10 m with or without assistive devices. Exclusion criteria are progressive neurological or rheumatological conditions, a diagnosed vestibular problem, current pain condition that significantly limits or obstructs everyday living, known uncorrected visual or hearing problems, not able to speak, understand, and read Danish, or cognitive impairment. Since executive function is related to falls [50–52], and the present study protocol requires the participants to wear accelerometers for several days and to recall fall episodes retrospectively (therefore reducing risk of bias), we decided to only include subjects without significant cognitive impairments. In cases where the experienced tester gauge the participant to be cognitively impaired, the participant is to complete a Mini-Mental State Examination (MMSE). Participants with a MMSE < 23 are to be excluded. Information on the intake of medications that might affect postural balance and/or physical mobility is not collected and thus not considered an exclusion criterion.

Participants satisfying the inclusion and exclusion criteria can be recruited in three different ways:

1. Through employees at activity centers in Aalborg Municipality. The employee contacts the participant directly at the center or by phone and hand out information material. Date, time and place for the baseline tests are arranged in collaboration with the project manager.
2. The employee gives the name and phone number of the participants to the project manager who then arranges the date, time and place for baseline testing with the participant.
3. Participants can make contact to the project manager directly by phone or e-mail. Information material is sent by e-mail or given verbally over the telephone to ensure that the participant fully understands what it takes to participate. Subsequently, an appointment for the time and place for completion of the baseline data is made.

**Fig. 1:**
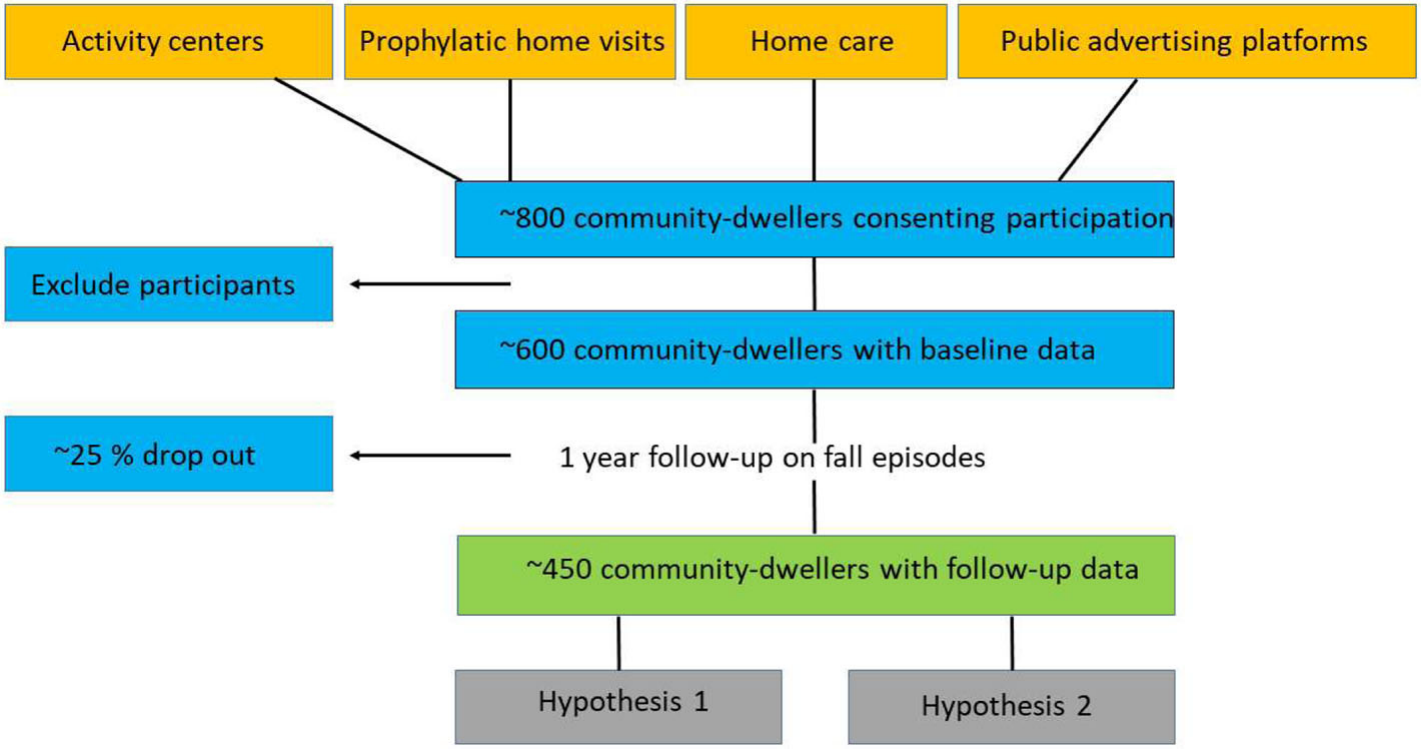
Illustration of the recruitment flow of the NOCfao study

The included participants are arranged in groups and baseline testing will take place at an activity center nearby their home. On this day, information regarding the study procedure is provided and informed written consent is obtained in order to make sure that the participants fully understand the requirements of participation. An overview of the recruitment procedure is illustrated in Fig. 1.

### Statistical analyses and sample size justification

Descriptive statistics (e.g., mean, standard deviations, and range) will be performed in order to describe the study population characteristics. Inferential statistics (e.g., t-tests and ANOVAS) will be applied to explore any significant differences between non-fallers, fallers and multiple fallers. Regression and correlation analysis will be undertaken to identify candidate variables (e.g., when combined and/or adjusted) that are significantly associated with falls. Relative risks will be calculated to quantify the association between candidate variables (physical activity and postural sway) as well as psychological factors, fall incidence, socio-demographic data, health information, physical tests, pain sensory profile, and risk of falls between groups. Estimates and confidence intervals will be reported accordingly.

All statistical analyses will be performed using SPSS Statistics software (IBM, Inc., Zurich, Switzerland), STATA (StataCorp, College Station, Texas, USA), Statistica (StatSoft, Inc. (2011). STATISTICA (data analysis software system), version 10. www.statsoft.com.) or MATLAB (The MathWorks Inc., Natick, Massachusetts, USA) and a significance level of 5% will be used for statistical significance. All future statistical reporting from the NOCfao study will follow the Strengthening the Reporting of Observational studies in Epidemiology (STROBE) statement [﻿47﻿].

The size of the study population was estimated based on previous studies on objective measures of physical activity (exposure) and self-reported fall incidence by telephone interview (outcome). Various Danish studies and cohorts have used diurnal recordings of physical activity with accelerometers [53, 54], including the NOMAD study and the DPhacto cohort. Depending on the specific aim of studies from these cohorts, the number of included participants varies from *n* = 198 [﻿55﻿], *n* = 457 [﻿56﻿] and up to *n* = 657 [﻿57﻿]. Among community dwelling older adults aged ≥65 years, approximately one third falls at least once a year [2, 6, 7]. The sample size in studies on fall incidence using telephone interviews have used samples of *n* = 326 and *n* = 331 participants [﻿58﻿, 59]. The assumed number of participants needed at various stages in the recruitment flow from the first contact to complete follow-up data are inspired and modified from the DPhacto protocol paper [﻿53﻿] and are illustrated in Fig. 1. Thus, based on previous studies on both the exposure and outcome, it is estimated that a sample of 600 participants is needed for baseline and 450 participants with complete baseline and follow-up data.

### Data collection

**Fig. 2:**
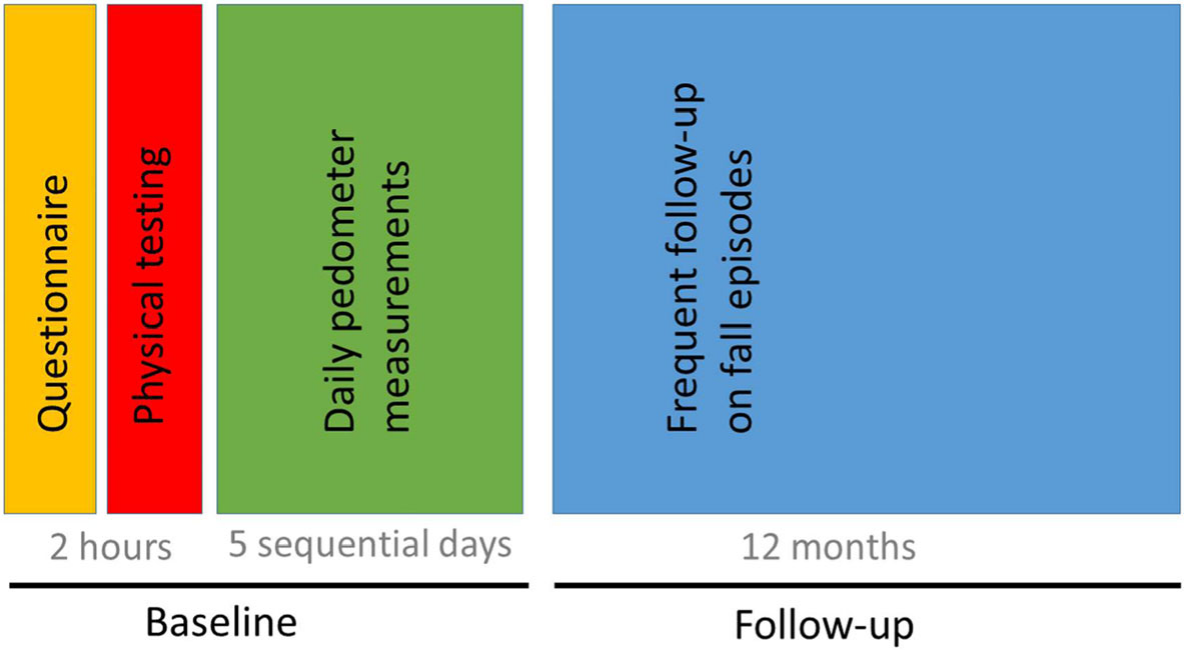
The NOCfao study timeline

The NOCfao-study is a prospective cohort study with a one-year follow-up of falls using frequent reports of fall incidence, see Fig. 2 and Table 2.

**Table 2:** Assessment overview of the timing and content in the NOCfao study

Domain	Type of Assessment	Notes	BL	DM	FU		Origin
Demographic data	Age	Self-reported	x				
	Gender	Self-reported	x				
	Height	Measured in centimeters	x				
	Weight	Digitally measured on forceplate	x				
	Marital status	Self-reported	x				
	Type of dwelling	Self-reported	x				
	Use of mobility aids	Self-reported	x				
	Use of homecare	Self-reported	x				
Health information	Chronic diseases	Self-reported	x				
	Number of prescribed medications	Self-reported	x				
	Fluid intake	Self-reported	x				
Physical activity	Objectively measured physical activity	Pedometer (Stepwatch3), Repeatedly over 5 days		x			[60, 61]
	Self-reported physical activity	IPAQ-elderly	x				[﻿62﻿]
Pain sensory profile	Muscle tissue	Pressure Pain Threshold	x				[﻿63﻿]
	Severity and duration of pain	Self-reported obtained by NRS	x				[﻿64﻿, 65]
	Location of pain	Body chart	x				[﻿66﻿]
Physical tests	Walking speed	10 m walking test	x				[﻿67﻿, 68]
	Muscle strengths in lower extremities	30-s sit-to-stand	x				[﻿69﻿]
	Muscle strengths in upper extremities	Seehan digital Hand grip dynamometer	x				[﻿70﻿, 71]
	Balance	Force plate measures	x				[﻿72﻿]
		Mini-BESTest	x				[﻿73﻿, 74]
Psychological factors	Anxiety of falling	Falls efficacy scale - International	x				[﻿75﻿]
Fall incidence	Fall incidence and fall circumstance	Phone-interview on a monthly basis. Prospective 1 year			x		[﻿76﻿]
	Fall history	Self-reported one-year history of falls	x				

Notes: *BL*: Baseline; *DM*: Diurnal measurements; *FU*: Follow-up

In order to collect baseline data, individuals included in the study are asked to:

1. Complete three questionnaires to describe: the characteristics of the cohort, health information, history of falls, anxiety of falling, and self-reported PA
2. Perform physical tests measuring strength in the upper and lower extremities, balance, and walking speed
3. Participate in assessment of pain sensitivity by recordings of pressure pain threshold (PPT)
4. Wear an ankle mounted pedometer (Stepwatch 3) for measuring activity for five consecutive days

Baseline data, application and use of the activity monitor are supervised by physiotherapy students under the guidance of an experienced physiotherapist. The students receive training through several workshops to ensure high reliability in the test procedure.

To monitor fall incidences, the project manager or students contact participants on a monthly basis by phone throughout the year after recruitment. The numbers of fall incidences during the previous 1 or 2 months are collected, and the circumstances surrounding the falls are registered. The baseline data will be collected between the autumn of 2016 and the end of autumn 2019, and follow-up data between the autumn of 2017 and 2020 (see Table 2).

Data in paper form collected at baseline are physically stored in locked cabinets in a room with an electronic lock complying with the Danish Data Protecting Agency and Institutions guidelines. All data will be entered electronically and stored in a secured database.

In terms of confidentiality data will be identified by a coded ID number. All records that contain names or other personal identifiers, such as locator forms and informed consent forms, will be stored separately from study records identified by code number.

### Physical activity

To monitor the physical activity levels among elderly, the Step Watch 3 Activity Monitor (SAM) is used. SAM is an ankle-worn pedometer and contains a custom sensor that uses a combination of acceleration, position, and timing to detect steps [﻿77﻿]. SAM is calibrated based on each participants height and gait pattern. The SAM has been deemed valid and reliable for monitoring step count at different walking speeds, especially for slow speeds [60, 61, ﻿78﻿], which is highly relevant for the current study. The step count is recorded over five consecutive days [﻿79﻿] with participants wearing the SAM during daytime. An activity log is provided so the participants can fill in non-wear pedometer periods.

In addition to the recorded step count, participants also complete a self-reported questionnaire: International Physical Activity Questionnaire, Elderly, Short Form (IPAQ-elderly). It contains four questions involving time spent 1) sedentary, 2) walking, 3) with moderate physical activities, and 4) with vigorous physical activities during the previous 7 days. IPAQ-elderly is a valid and reliable questionnaire [﻿62﻿, 80]. As the questionnaire does not exist in a Danish version, the Swedish version is used. Sweden and Denmark are both Scandinavian countries with similarities regarding the elderly population, culture, healthcare system, and language [81–83]. As recommended, the test-person receives guidance from an experienced tester when answering the questionnaire [﻿84﻿].

### Fall incidence

Fall history of the participants are measured retrospectively as a 12-month recall of falls at baseline. Prospective falls are ascertained over a 12-month period by monthly phone calls with the purpose of determining both the number of falls, why the fall occurred, where it occurred, and when during the day the fall incidence happened. Falls are listed as number of falls and expressed in groups: fallers, non-fallers and multiple fallers along with fall rate per person in a year. This is in accordance with international recommendations [﻿84﻿].

### Socio-demographic data

The socio-demographic data describing age, gender, marital status, type of dwelling, use of mobility devices, and use of home-care are collected. These data are registered at baseline through a self-reported questionnaire. Weight and height are measured objectively, using the Tanita Digital Scale for weight measured in kilograms and Tanita Leicester Height measure system for height measured in centimeters. All measures are approximated to the next integer.

### Health information

Health information including chronic disease, the number of prescribed medication and daily fluid intake are likewise registered at baseline through a self-reported questionnaire. The question concerning chronic disease is two-item (i.e., yes or no response possibility) with the possibility to specify the diagnoses. The question concerning the use of prescription medication is also two-item (i.e., yes or no response possibility, and if yes, the number of medications). Over the counter medicines are not accounted for in this study. Daily fluid intake (i.e., water, soft drink, coffee, tee, juice, milk and fruit syrup) is five-item (i.e., 0-½, ½-1, 1–1½, 1½-2, and > 2 l response possibility).

### Physical tests

#### Walking speed

Self-selected walking speed is associated with fall status [67]. A 10 m walking test (walk timed section), includes an acceleration and deceleration zone of 5 m each, is used as a measure for walking speed. This test is found to be valid and reliable for community-dwelling older adults [85]. The participant is standing still in the anatomical neutral position and asked to walk straight forward at a self-selected walking speed. The timed walking distance of 10 m, measured in seconds, is only known by the tester, and the participant is instructed to walk to an endpoint further than 10 m. This is repeated three times, with a 20 s pause between each trial. The fastest value in m/s is noted as final score. The test can be performed with a walking-devise if necessary. The standardization of the Danish version implicates a static start, where other versions have a 5 m. acceleration and deceleration zone. However, a study by Lindholm et al. has shown that there does not appear to be a need for using an acceleration distance among people with mild Parkinson’s disease [68]. The participants in this study had an average comfortable walking speed equivalent to 1,15 m/s, which is quite similar to the average walking speed for community-dwelling older adults [85].

#### Muscular strength

Other studies have found an association between handgrip strength and risk of falling [70, 71] and therefore it is relevant to explore if a similar association is present in a Danish population. Grip strength is measured with Saehan Digital Hand-Dynamometer. The participant is asked to sit in a chair with the upper arm along the side of the body and elbow flexed at 90 degrees. The participant is instructed to squeeze the handle as hard as possible for 3 s, followed by a 30 s pause between each test. Both arms are tested three times and the highest score for each arm is extracted for data analysis purpose.

A thirty seconds sit-to-stand test is used as a measure for lower limb strength. The participant starts by sitting on a chair with armrests, with the upper body free of the backrest and arms crossed in over the chest. The subject is instructed to perform as many sit-to-stand movements as possible within a 30 s timeframe. This test is found reliable and valid as a strength measure for lower limb [69, 86, 87] and an important predictor for risk of falling with a cut-off score of eight repetitions [88–90].

#### Balance

To explore the postural balance of the elderly, a clinical test battery (Mini BESTest) and a force platform (AMTI®, model: Dual-top AccuSway, Watertown, Massachusetts, USA) are used. The Mini BESTest employs 14 different physical tasks challenging both the proactive and reactive balance of the elderly. Each item has a score from 0 to 2 (2 = best score), with a maximum score of 28 points. This test is known to be both valid and reliable to community-dwelling older adults with balance deficits [91, 92], with a cut-off score of 16 as a predictor for risk of falling [74].

Research has shown an association between increased postural instability and risk of falling [93]. The force platform (acquisition rate: 50 Hz, resolution 12-bit) will measure the forces and moments applied by the participants during all postural tasks. The center of pressure (CoP) position in time will be estimated by the forces moments via a custom-made script in MATLAB. The CoP data will be filtered with a zero-lag low-band pass filter (10 Hz) and possible variables such as CoP displacement and velocity (among others) will be extracted to evaluate the participants postural sway and stability in five different tasks: 1) standing on the platform and swaying forwards-backwards and from side to side without changing the base of support, 2) stepping down from the force platform, 3) stepping up onto the force platform, 4) standing still with eyes open, and 5) standing still with eyes closed [94].

### Pain sensory profile

It is known that the occurrence of musculoskeletal pain is significantly associated with the risk of falls amongst community-dwelling older adults [95, 96]. Therefore, assessing the sensitivity of pain mechanisms is a relevant outcome measure to account for in this population. A handheld pressure algometer (*Somedic, Hørby, Sweden*) mounted with a 1 cm^2^ probe enclosed in a disposable latex cover is used to assess Pressure Pain Threshold (PPT). By random selection, the probe is placed over either left or right side of the shoulder and anterior on crus, equivalent to the most protruding part of musculus deltoideus and tibialis anterior. PPT is defined as the first time the pressure is perceived as painful, and at this point the participant pushes a button wired to the algometer which will then record the pressure. The pressure will be gradually increased at a rate of 30 kPa/s until PPT is reached. Each measurement will be conducted three times during each session and the mean will be used for further analyses. In addition, the participants’ pain experience is covered in relation to duration (ordinal scale from 1 to 7 days to ≥6 month), severity (Numeric Pain Rating Scale), and location (Body Schema).

### Psychological factors

#### Fear of falling

Falls Efficacy Scale International (FES-I) is a self-report questionnaire revealing concerns regarding falls amongst older adults. The questionnaire is developed in Europe and translated into 14 different languages including Danish [75, 97]. It is a widely accepted tool for assessing concerns about falling, developed by the Prevention of Falls Network Europe (ProFaNE). It contains 16 items scored on a scale from 1 to 4 (1 = not at all concerned, 4 = very concerned), and is found to be valid and reliable to older adults, with a cut-off score ≥ 23 for high concern [98].

## Discussion

It is the authors’ belief that the NOCfao study will provide a distinctive and clinically applicable method to fall risk assessment for community-dwelling older adults. The main aim of this paper is to describe the methods and foundation of the NOCfao study. If the main study hypotheses of this prospective, observational cohort study are accepted, this will highlight the importance of physical activity and impaired postural stability in relation to falls among community-dwelling older adults. Applying this knowledge in this population may contribute to a reduction in the number of falls, which will benefit the community-dwelling older adults, the family, the community, and reduce the societal costs.

This study encompasses various important strengths to supplement to the present-day understanding on the association between physical activity and impaired postural stability and the number of falls among community-dwelling older adults. For objective measures of step monitoring, the SAM is small and light-weighted and is reported to be well-tolerated by older patients [78]. It does not interfere with the community-dwelling older adults’ daily life, nor does it provide any information to the participants that may introduce sudden changes in motivation and thereby the level of PA. This is therefore expected to reduce the risk of under- or overestimation. The measures of PA will contain several days of recordings, which will advance the authentic representation of PA in community-dwelling older adults. Lastly, the objective measures using SAM have been shown to be valid and reliable in populations of community-dwelling older adults [60, 78], but SAM has not yet been introduced in cohorts on fall risk assessment. The prospective monthly follow-up on number of falls and the circumstances they occur in, reduces the risk of recall bias and allows for stratified analyses on specific type of fall incidence.

### Limitations and methodological considerations

We recognize that the NOCfao study contains limitations. First, the objective measures of PA are represented only by the recorded step count and the walking speed obtained from the 10 m walking test. Even though these two methods are valid, reliable, and associated with fall status [38, 67], they only portray a narrow aspect of PA. Therefore, a self-reported questionnaire (i.e. IPAQ-elderly) encompassing information on time spent 1) sedentary, 2) walking, 3) with moderate physical activities, and 4) with vigorous physical activities during the last 7 days are also applied. Nevertheless, these self-reported measures of PA can be subject to bias, given their link to elements such as community-dwelling older adults’ tendency to overestimate or underestimate the level of PA, recall errors, misunderstanding the question format, social desirability in front of an interviewer, and lower educational level [84, 99–101]. The interaction remains uncertain with respect to the relationship between PA and the prospective one-year follow-up on falls. Further, the recruitment process may initiate a selection bias among the community-dwelling older adults included in this study. In other words, the participating community-dwelling older adults included in this study may be resourceful and motivated in terms of their ability to locate and respond to the advertising platforms, and perform better at a baseline test as well as wearing SAM, keeping a diary, and being willing to provide information throughout a 12-month period. An important non-physical risk factor, executive function [102] is currently not included in the present protocol and should be addressed in the future studies.

This study will add to the present-day understanding of the association between PA and impaired postural stability and the number of falls among community-dwelling older adults, providing valid and reliable information on the relationship and its significance among community-dwelling older adults.

## Data Availability

Data sharing is not applicable to this article as data collection is not yet finished. Once the data collection is finished and the data has been analyzed and published, the analyzed data will be available upon reasonable request.

## Abbreviations

ProFaNE: Prevention of Falls Network Europe
PA: Physical activity
NOMAD: New method for Objective Measurements of physical Activity in Daily living
DPhacto: Danish PHysical ACTivity cohort with Objective measurements
MMSE: Mini-Mental State Examination
NOCfao: the ≥65 years NOrthern jutland Cohort of Fall risk Assessment with Objective measurements
SAM: Step Watch 3 Activity Monitor
FRPT: Fall risk prediction tool
IPAQ-elderly: International Physical Activity Questionnaire, Elderly, Short Form
NRS: Numeric rating scale
FES-I: Falls efficacy scale - International
PPT: Pressure pain threshold
SPIRIT: Standard Protocol Items: Recommendations for Interventional Trials
BL: Baseline
DM: Diurnal measurements
FU: Follow-up
UCN: University College of Northern Denmark
NTNU: Norwegian University of Science and Technology
SMI: Center for Sensory-Motor Interaction
